# Supplementary material for: Intranasal Anti-CD3 Antibody Treatment Attenuates Post–COVID Neuroinflammation and Enhances Hippocampal Neurogenesis and Cognitive Function in Mice
Source: bioRxiv. 2026 Apr 16:2026.04.07.716934. Originally published 2026 Apr 8. Preprint. [Version 2] doi: 10.64898/2026.04.07.716934 (PMC13082027; doi:10.64898/2026.04.07.716934)
Supplement: Supplementary file 1 [file NIHPP2026.04.07.716934v2-supplement-1.pdf]

bioRxiv preprint doi: https://doi.org/10.64898/2026.04.07.716934; this version posted April 16, 2026. The copyright holder for this preprint (which was not certified by peer review) is the author/funder, who has granted bioRxiv a license to display the preprint in perpetuity. It is made available under a CC-BY 4.0 International license.

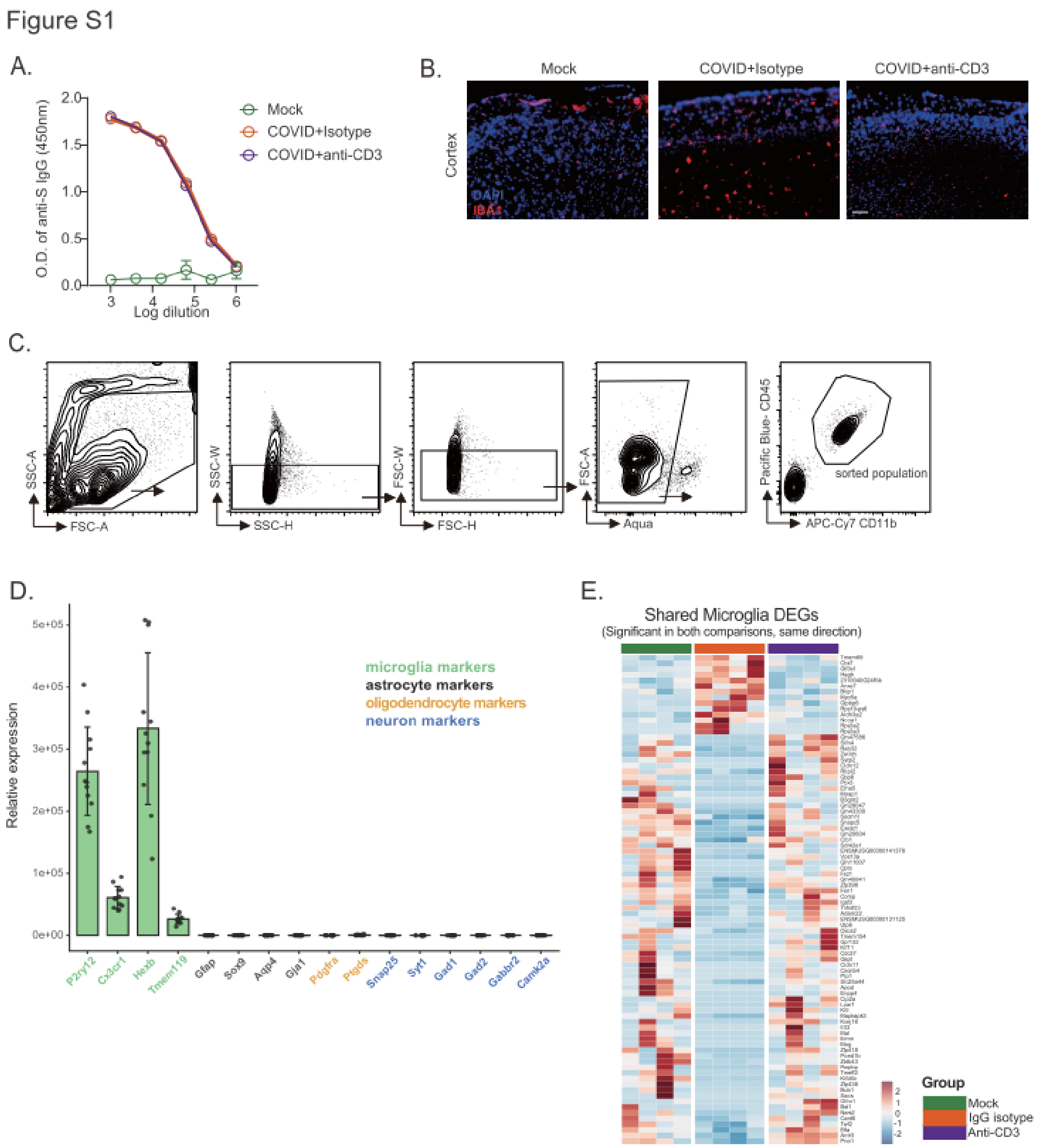

Figure. S1 Additional data of early nasal anti-CD3 treatment in mild COVID mice, related to Figure1 & 2. (A) Serum antibodies were measured against spike protein using an ELISA, n=6-7 mice per group. (**B)** Quantification and confocal micrograph of microglia (IBA1+) in the cortex after 4 weeks isotype or anti-CD3 antibody administration in mild COVID or mock mice; n=6-7 mice per group. Data shown as mean ± SEM; each dot represents an individual mouse. *p <0.05, ** p <0.01, ***p <0.001, ****p <0.0001 (one way ANOVA followed by Tukey's multiple comparison test). **(C)** Representative gating strategy to isolate microglia for bulk RNAseq. **(D)** Relative expression of cell type-specific markers in sorted microglia. **(E)** Heatmap of shared microglial DEGs significant in both comparisons (Mock vs IgG isotype and anti-CD3 vs IgG isotype) and changing in the same direction. n=4 or 5 mice per group.

bioRxiv preprint doi: https://doi.org/10.64898/2026.04.07.716934; this version posted April 16, 2026. The copyright holder for this preprint (which was not certified by peer review) is the author/funder, who has granted bioRxiv a license to display the preprint in perpetuity. It is made available under a CC-BY 4.0 International license.

Figure S2

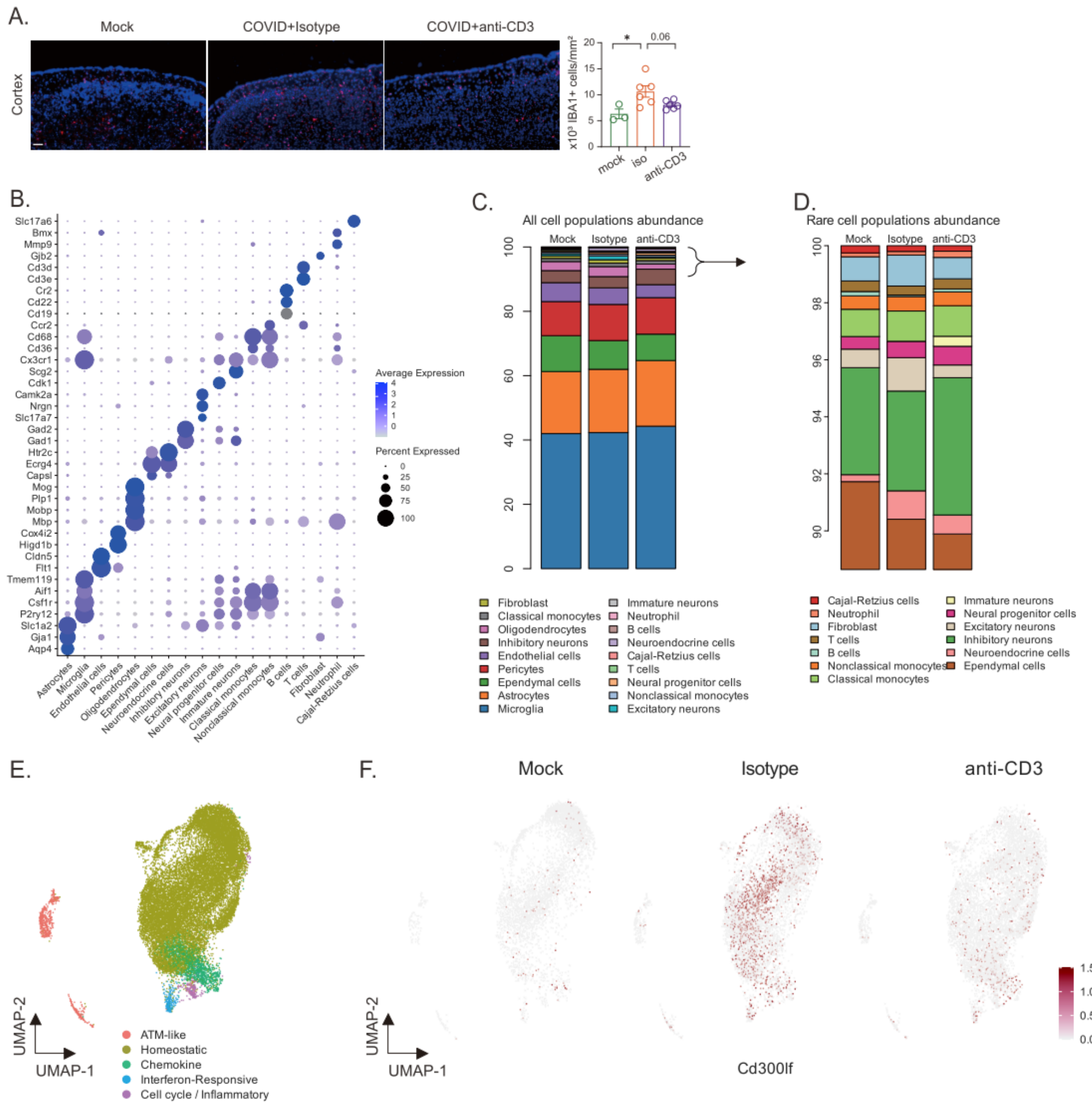

Figure. S2 Additional data of late nasal anti-CD3 treatment in mild COVID mice, related to Figure3 & 4. (**A**) Quantification and confocal micrograph of microglia (IBA1+, red) in the cortex after 4 weeks isotype or anti-CD3 antibody administration in mild COVID or mock mice; n=3-7 mice per group. Data shown as mean ± SEM; each dot represents an individual mouse. *p <0.05, ** p <0.01, ***p <0.001, ****p <0.0001 (one way ANOVA followed by Tukey's multiple comparison test) .(**B**) Dot plot showing expression of cell type marker genes used in the annotation the clusters shown in figure 4C and 4D. **(C)** Distribution of total cell subsets in the scRNAseq and **(D)** distribution of the least abundant cell types. **(E)** UMAP plot of microglia

bioRxiv preprint doi: https://doi.org/10.64898/2026.04.07.716934; this version posted April 16, 2026. The copyright holder for this preprint (which was not certified by peer review) is the author/funder, who has granted bioRxiv a license to display the preprint in perpetuity. It is made available under a CC-BY 4.0 International license.

colored by clusters. **(F)** UMAP plots as in (E) colored by expression level of Cd300lf.
